# Dementia in 50 Years and Older Patients from the Psychiatry Out Patient Department of a Tertiary Care Center: A Descriptive Cross-sectional Study

**DOI:** 10.31729/jnma.6907

**Published:** 2021-10-31

**Authors:** Mohan Belbase, Jyoti Adhikari

**Affiliations:** 1 Department of Psychiatry, Nepalgunj Medical College Teaching Hospital, Kohalpur, Banke, Nepal; 2 Department of Pediatrics, Nepalgunj Medical College Teaching Hospital, Kohalpur, Banke, Nepal

**Keywords:** *alcohol*, *alzheimer's*, *dementia*, *prevalence*

## Abstract

**Introduction:**

Dementia is a chronic and progressive syndrome due to disease of brain. Alzhemeir's disease is the most common cause of dementia. There are very few studies regarding prevalence of dementia in Nepal. The objective of this study is to find the prevalence of dementia in 50 years and older patients from the psychiatry out patient department of a tertiary care center.

**Methods:**

A descriptive cross-sectional study was done in patients attending to psychiatry outpatient department over one year from May 2020 to April 2021. Ethical approval was taken from the Institutional Review Committee (Reference number: 745/077-078). The data were assessed using Statistical Package for Social Sciences version 20. Point estimate at 95% Confidence Interval was calculated along with frequency and proportion for binary data.

**Results:**

Out of 1332 patients, 52 (3.9%) (95% Confidence Interval=2.86-4.93) patients had dementia in which 30 (57.7%) are male while 22 (42.3%) are female. Mean age of study population is 70.12±11.21 with age range of 50 years to 88 years. We found 29 (55.8%) moderate, 18 (34.6%) severe and 5 (9.6%) mild type of dementia. We had 34 (65.3%) Alzheimer's followed by 16 (30.8%) vascular and 2 (3.9%) others types of dementia.

**Conclusions:**

This study concludes that the prevalence of dementia in 50 years and older patients is similar in comparison to other studies done in similar settings.

## INTRODUCTION

Dementia is a progressive disease with disturbance of multiple higher cortical functions.^1^ It may be caused by toxins or metabolites, infection, degenerations but age is the leading risk factor for dementia.^2^ The cost of disease burden is huge as there are more elderly people living due to improved health care facilities.^3^ In 2010, it was estimated there are 35.6 million people living with dementia worldwide.^4^ In Nepal, there may be over 135000 people with dementia.^5^ There are certain modifiable risk factors while age and ethnicity are non modifiable. Dementia results from interactions of comorbidities, genes and the environment.^6,7^

There is dearth of studies about different types, comorbidity and severity of dementia in the developing countries including Nepal. Moreover, study of dementia in 50 years and older is even less as most of the studies focus on elderly population.

The objective of this study is to find the prevalence of dementia in 50 years and older patients from the psychiatry out patient department of a tertiary care center.

## METHODS

This is a descriptive cross-sectional study done in patients attending to psychiatry outpatient and inpatient department in Nepalgunj Medical College, Kohalpur. Ethical clearance was taken from Institutional review committee (Ref.745/077-078). Out of total 1332 (516 male and 816 female) patients aged 50 years or more visited to psychiatry OPD from May 2020 to April 2021 in one year, suspected cases were first selected by using brief mental state examinations. Convenience sampling was done and those who gave written consent were included in the study. In patients who were unable to give written consent, consent was taken from immediate guardian and caretakers. Patients having history of alcohol use (not active but history of alcohol abuse or dependence) were included. Patients who did not give consent, who were actively dependent on any psychoactive substance, had head injury, delirium, psychosis and younger than 50 years were excluded from the study. The sample size was calculated using the formula,

n = Z^2^ × p × q / e^2^

  = 1.96^2^ × 0.0129 × (1-0.0129) / (0.01)^2^

  = 3.8416 × 0.0129 × 0.9871/0.0001

  = 489

Where,

n = required sample size
Z = 1.96 at 95% Confidence Interval
p = prevalence of dementia in 50 years and older in reference population 1.29%^7^
q = 1-p
e = margin of error, 1%

Adding 10% non-response rate we get 538. However, 1332 samples were taken. Following data collection tools were used, the ICD 10 classification of mental and behavioral disorders, diagnostic criteria for research, Semi-structured pro forma and MMSC check list (Nepali version). Data was collected in pro forma developed by the department of psychiatry. The data were analyzed using Statistical Package for Social Sciences version 20 and analyzed and presented in tables with frequencies and percentages.

## RESULTS

Out of 1332 patients, the prevalence of dementia in 50 years and above was found to be 52 (3.9%) (95% Confidence Interval = 2.86-4.93) of which 516 (38.73%) were male 816 (61.26%) were female. Suspected cases were selected after initial brief mental state assessment and further interviewed. After a through screening using MMSE and ICD-10 diagnostic guidelines, 52 patients found to have dementia. Thus the prevalence rate of dementia in 50 years and above was 2 (3.9%).

Out of 52 dementia patients 30 (57.7%) were male while 22 (42.3%) were female. Age ranges from 5088 years, 70.13 ± 11.21. Majority 36 (69.2%) were illiterate while 16(30.8%) were literate. Previous history of alcohol use was found in 20 (38.5%) of study population. Depression was present in 17 (32.7%) of patients while cerebro vascular accident (CVA) was present in 16 (30.8%).

Similarly as per the MMSE score categorizations, most common cognitive dysfunction was moderate 29 (55.8%) followed by severe 18 (34.6%) while mild cognitive dysfunction was present in 5 (9.6%).

Results are presented in the number and percentages in table that shows the gender, age, literacy status, history of alcohol use, comorbid depression, comorbid CVA and severity of dementia with MMSE scoring (Table 1).

**Table 1:** Showing gender, educational level, history of alcohol use, depression, physical comorbidity (CVA) with severity of dementia in the patients having dementia.

Characteristics	Categories	No of Patients n (%)
Gender	Male	30 (57.7)
	Female	22(42.3)
Education level	Literate	16 (30.8)
	Illiterate	36 (69.2)
History of alcohol use	Used	20 (38.5)
	Not used	32 (61.5)
Depression	Present	17 (32.7)
	Absent	35(67.3)
Physical comorbidity	Present	16 (30.8)
(CVA)	Absent	36 (69.2)
MMSE score categorization	Severe	18 (34.6)
	Moderate	29 (55.8)
	Mild	5 (9.6)

In age range of dementia patients, most common rangewas 70-79 years that includes 22 (42.3%) followed by80 years or more 12 (23.1%), 50-59 years 10 (19.2%)with lowest being 60-69 years 8 (15.4%) (Table 2).

**Table 2:** Showing the age range with number and percentage of cases having dementia (n = 52).

Age range	n (%)
50-59	10 (19.2)
60-69	8 (15.4)
70-79	22 (42.3)
≥80	12 (23.1)
Total	52 (100)

Regarding the types ofdementia,dementia due to Alzheimer's was 34 (65.3%) followed by vascular 16 (30.8%) with the least being other causes including HIV infection 2 (3.9%) (Table 3,4).

**Table 3:** Showing types of dementia with their frequencies.

Dementia type	n (%)
Alzheimer's	34 (65.3)
Vascular	16 (30.8)
Others (HIV)	2 (3.9)

**Table 4:** Showing one-way ANOVA for comparision of age for others to compare gender, age, educational level, history of alcohol use, depression and CVA.

Characteristics	Categories	MMSE Score		
		Severe n (%)	Moderate n (%)	Mild n (%)
Gender	Male	14 (46.7)	13 (43.3)	3 (10.0)
	Female	4 (18.2)	16 (72.7)	2 (9.1)
Education level	Literate	2 (12.5)	12 (75.0)	2 (12.5)
	Illiterate	16 (44.4)	17 (47.2)	3 (8.3)
History of alcohol use	Used	11 (55.0)	7 (35.0)	2 (10.0)
	Not Used	7 (21.9)	22 (68.8)	3 (9.4)
	Present	5 (29.4)	10 (58.8)	2 (11.8)
Depression	Absent	13 (37.1)	19 (54.3)	3 (8.6)
Physical comorbidity (CVA)	Present	3 (18.8)	12 (75.0)	1 (6.2)
	Absent	15 (41.7)	17 (47.2)	4 (11.1)

## DISCUSSION

The prevalence of dementia in patients 50 years or above in our study is 3.9%. This is comparable to study entitled annual period prevalence and risk factors of dementia among older Jordanian hospitalized patients in which the general annual period prevalence of dementia for people older than 50 years was 1.29%.^7^ However our finding is not consistent with another similar study from a referral hospital of eastern Nepal where the prevalence rate of Dementia for patients aged 60 years or more in hospital psychiatry OPD was 11.4%.^8^ This contrast may be due to inclusion of 50 years or more patients in our study in which dementia is not much remarkable. Also, it may be the fact that people in western Nepal have lack of awareness with regard to cognitive problems including dementia.

Regarding the age groups, 22 (42.3%) were of age group 70-79 followed by 12 (23.1 %) in 80 and more; 10 (19.2%) in 50-59 years and 8 (15.4%) in 60-69 age brackets. Dementia progresses with age but this finding of more cases in age bracket of 70-79 may be due to lower life expectancy in Nepalese population with respect to developed and western countries. Also, more advance the age of a person, all the memory and behavioral problems are regarded normal for that age than considering a problem in our society and may not have been brought to hospital.

Regarding the education level, literate are 16 (30.8%) while illiterate are 36 (69.2%). There is a history of alcohol use in 38.5% of study population while majority had no history of alcohol use.

Depression and CVA very common mental and physical comorbidity in patient with dementia and we have almost one third 32.7% and 30.8% patients respectively having comorbid depression and CVA during the time of check ups for dementia. Depression itself can cause pseudodementia which may further exacerbate the remaining cognitive resources in the individual suffering from dementia. Comorbidity of depression in dementia in our study is in line with another study in 40 dementia patients from India regarding behavioral and psychological symptom of dementia in which there were 37% of patients with affective disturbances.^9^ In another study in 183 person of age 60 or above from a rural community from West Bengal, 61% had mental illness and the majority of affected person had depression.^10^ In another study from eastern Nepal, depression was found in 36.7% of dementia patients.^8^

We found 29 (55.8%) moderate, 18 (34.6%) severe and 5 (9.6%) mild type of dementia as per the finding from MMSE.

As per our study finding, we have 34 (65.3%) Alzheimer's followed by 16 (30.8%) vascular and 2 (3.9%) others types of dementia. The type of dementia in our study is almost similar to one study from India in which there were 54% people having Alzheimer's dementia, followed by vascular (39%) and others (7%).^11^ In another study regarding prevalence of dementia in urban Indian population, there were 65% had Alzheimer's dementia followed by vascular (22%) with increasing age being associated with higher prevalence of dementia.^12^

Use of psychoactive substance including alcohol is said to be responsible for of cognitive disturbances. One review article regarding alcohol use and dementia concludes that reducing heavy alcohol use may be an effective dementia prevention strategy.^13^ Though age cannot be modified, control in consumption of alcohol can be directly or indirectly help in prevention or progression of dementia.

## CONCLUSIONS

The prevalence of dementia in 50 years and older age group was found to be similar to other studies. Hence, dementia is prevalent not only in elderly population but also in 50 years and older age group. Moderate dementia is more common in these age groups followed by severe and mild types.
